# Temporal accumulation and localization of isoflurane in the C57BL/6 mouse and assessment of its potential contamination in ^19^F MRI with perfluoro‐crown‐ether‐labeled cardiac progenitor cells at 9.4 Tesla

**DOI:** 10.1002/jmri.25564

**Published:** 2016-12-19

**Authors:** Christakis Constantinides, Mahon L. Maguire, Leeanne Stork, Edyta Swider, Mangala Srinivas, Carolyn A. Carr, Jurgen E. Schneider

**Affiliations:** ^1^ Department of Cardiovascular Medicine, Nuffield Department of Medicine, Radboud University Medical CenterRadboud UniversityNijmegenThe Netherlands; ^2^ Department of Physiology, Anatomy, and GeneticsUniversity of OxfordOxfordUnited Kingdom; ^3^ Department of Tumor ImmunologyRadboud University Medical Center, Radboud UniversityNijmegenThe Netherlands

**Keywords:** cell labeling, ^19^F MRI, isoflurane localization, contamination

## Abstract

**Purpose:**

To assess the uptake, accumulation, temporal stability, and spatial localization of isoflurane (ISO) in the C57BL/6 mouse, and to identify its potential interference with the detection of labeled cardiac progenitor cells using ^19^F MRI/MR spectroscopy (MRS).

**Materials and Methods:**

Objectives are demonstrated using (a) in vitro ISO tests, (b) in vivo temporal accumulation/spatial localization C57BL/6 studies (n = 3), and (c) through injections of perfluoro‐crown‐ether (PFCE) labeled cardiac progenitor cells into femoral muscle areas of the murine hindlimb post‐mortem (n = 1) using ^1^H/^19^F MRI/MRS at 9.4 Tesla. Data were acquired using double‐gated spoiled gradient echo images and pulse‐acquire spectra. For the in vivo study, the temporal stability of ISO resonances was quantified using coefficient of variability (CV) (5 min) estimates.

**Results:**

Two ISO resonances were observed in vivo that correspond to the ‐CF_3_ and ‐OCHF_2_ moieties. CV values ranged between 3.2 and 6.4% (‐CF_3_) and 6.4 and 11.2% (‐OCHF_2_). Reductions of the ISO dose (2.0 to 1.7%) at 80 min postinduction had insignificant effects on ISO signals (*P* = 0.23; *P* = 0.71). PFCE‐labeled cells exhibited a resonance at −16.25 ppm in vitro that did not overlap with the ISO resonances, a finding that is confirmed with MRS post‐mortem using injected, labeled cells. Based on ^19^F MRI, similar in vivo/post‐mortem ISO compartmentalization was also confirmed in peripheral and thoracic skeletal muscles.

**Conclusion:**

Significant ISO accumulation was observed by ^19^F MRS in vivo with temporally stable signals over 90 min postinduction. ISO effects on PFCE labels are anticipated to be minimal but may be more prominent for perfluoropolyether or perfluorooctyl bromide labels.

**Level of Evidence**: 1

**Technical Efficacy**: Stage 1

J. MAGN. RESON. IMAGING 2017;45:1659–1667

Noninvasive imaging and tracking of labeled cells in vivo and their functional impact has taken an increasing role in recent years.^1^, ^2^, ^3^ Specifically, nanoparticles (NPs) containing perfluorocarbons (PFC) have enabled direct tracking and quantification of labeled cell populations using ^19^F MR spectroscopy (MRS) and MRI.^4^, ^5^ Correspondingly, labeling and in vivo MRI tracking of cells has been applied in small animal models of neurovascular disease,^4^, ^6^, ^7^, ^8^ exhibiting potential for translational work.^9^

Most preclinical studies are now routinely conducted using inhalational isoflurane (ISO),^10^, ^11^, ^12^, ^13^ the most commonly used volatile anesthetic owing to its minimal cardio‐depressive effects.^12^ Isoflurane (CF_3_CH_2_ClOCHF_2_) contains two groups (moieties) of ^19^F atoms, which give rise to two peaks (with chemical shifts of ‐4 and ‐10.3 ppm with respect to CFCl_3_) in the NMR spectrum. These peaks may interfere with the detection of fluorinated agents in vivo (chemical shift range of ‐126.6–9.1 ppm with respect to CFCl_3_ and ‐50–86 ppm with respect to trifluoroacetic acid [TFA]) due to potential spectral overlaps. Furthermore, despite the existence of numerous prior ^19^F MRI studies on the uptake and clearance characteristics of ISO in the brain in vivo,^14^, ^15^, ^16^, ^17^ the localization of ISO in cardiac thoracic and peripheral skeletal tissues, its bio‐distribution, accumulation, kinetics, and possible interference with ^19^F labels has not been investigated in detail. Additionally, only a few reported cardiovascular applications of fluorinated agents exist,^18^, ^19^ and investigations into the possible implications of the use of ISO have been speculative.^20^

Specifically, Flögel et al.^18^ and van Heeswijk et al.^19^ have, respectively, demonstrated successful cardiac and vascular ^19^F MR imaging in the mouse thorax following injection of PFC emulsions into the venous circulation. While Flögel et al.^18^ do not discuss the impact of the presence of ISO on the imaging of ^19^F emulsions, van Heeswijk et al.^19^ have shown minimal ISO accumulation of the anesthetic in the thoracic vascular space, and have quantified the relative spectral shifts from the injected agents. More recently, Fox et al.^20^ have argued that the use of ISO should have a minimal impact on the ^19^F MR images of PFC agents, and have suggested the use of selective radiofrequency (RF) excitation to ameliorate any potential issues; however, their discussion is speculative and lacks direct experimental evidence.

The scope of this study is generalized on the accumulation of ISO (in striated skeletal and cardiac muscles). It extends beyond the two prominent prior ^19^F MRS/MRI publications that focused on cardiovascular applications,^18^, ^19^ whereby direct injections of labeled emulsions were achieved by means of the intravenous route. Their reported approach^18^, ^19^ exhibits bio‐distribution, delivery, temporal dynamics, and label dilution differences, compared with current state‐of‐the‐art schemes using either direct injections (adopted strategy in this study), or scaffold‐based, ^19^F labeled, cardiac progenitor stem cell administrations.

Therefore, the objectives of this study are to: (a) determine the spatial localization of ISO in the thoracic and peripheral skeletal muscle areas of the C57BL/6 mouse, and quantitatively assess its uptake, accumulation, and temporal stability within a period of 90 min postinduction, including its potential accumulation in the ventricular blood pool, and myocardium, (b) define the spectral characteristics of the ISO moieties in vivo, (c) identify the spectral characteristics of extensively used ^19^F labels, and assess their potential spectral overlaps and interference with respect to ISO, including the extensively used perfluoro‐crown‐ether (PFCE) label, using ^19^F MRS/MRI.

## Materials and Methods

### Animal Ethics

All procedures were approved by the local ethical review committee (U. Oxford), and conformed to the Animal (Scientific Procedures) Act 1986 (UK) incorporating European Directive 2010/63/EU.

### MRI

All experiments were conducted on a 9.4Tesla (T) Agilent scanner equipped with a DirectDrive console and a 1 mT/m actively shielded gradient set (internal diameter = 60 mm) (Agilent Technologies, Santa Clara, CA).

#### Radiofrequency Coils

An eight‐rung, low‐pass quadrature birdcage coil (internal diameter = 34 mm), was constructed in‐house, tuned and matched at 375.8 MHz, and used for ^1^H/^19^F MRS/MRI. The broad frequency response of the coil permitted imaging on both the ^1^H and ^19^F nuclei. Post‐mortem imaging was conducted using a home‐built 80 × 40 mm^2^ butterfly coil.

#### Pulse Sequences

An autoshim method was used for in vivo mouse shimming on the ^1^H resonance, as described previously.^21^ In vivo ^1^H and ^19^F MR images were acquired in mice (thoracic area) using a segmented *k*‐space, prospectively electrocardiograph‐ (ECG‐) and respiratory‐gated (double‐gated) spoiled gradient echo (SPGR) sequence. A three‐dimensional (3D) SPGR sequence (ungated) was used for the post‐mortem imaging (hind‐limb skeletal muscle areas).

For MRS, the temporal evolution of ISO accumulation was monitored using nonlocalized, fully relaxed, pulse‐acquire spectra.

#### Phantom Studies

a) Aqueous sodium fluoride (NaF) phantoms at 50 mM [Sigma‐Aldrich, UK] (T_1_ = 1564 ms; T_2_ = 1257 ms) were used as reference markers in ISO studies for calibration and quantification. b) Pulse‐acquire spectra were collected (repetition time [TR] = 10 s; flip angle = 90 °; bandwidth [BW] = 10–20 kHz; number of excitations [NEX] = 1–10; 512–8192 points) using phantoms containing: (1) liquid ISO (ISO Flo, 100% w/w, Abbott Laboratories, UK), (2) PFC NPs (3.8–10 mg/mL) emulsified in deionized water and mixed in cell media solution (IMDM, Thermo Fisher Scientific, UK), and (3) 25 mM TFA, separately or in combination with NPs and PFCE‐labeled cells (TR = 20 s; flip angle = 90 °; NEX = 4; 512 points; BW = 20 kHz).

### Cells

#### Nanoparticle Formulation

Particles were synthesized in accordance to previously published methodologies.^22^, ^23^ In brief, nanoparticles (∼200 nm in diameter) with entrapped PFCEs were synthesized using poly(lactic‐co‐glycolic acid) (PLGA). All particles were washed extensively with distillated water and lyophilized for 3–4 days. Prior ^19^F MRS characterization of PFCE NPs has confirmed a single spectral peak at ‐16.00 ppm (with respect to TFA).

#### Cell Isolation

Cardiac progenitor cells comprising either collagenase‐trypsin (CT) or cardiosphere‐derived cells (CDC)] were isolated from adult, C57BL/6, GFP positive, mouse atria, using standard protocols.^24^, ^25^

#### Labeling

Cells were subsequently incubated with PFCE‐containing NPs (loading concentration of 10 mg/ml/million cells) for approximately 24 h before trypsinization, isolation, and washing. Cell pellets were suspended and maintained in IMDM media and were used for MRI, flow cytometry, or confocal microscopy (after fixation in 2% paraformaldehyde solution). Successful labeling was confirmed with a CyAn ADP flow cytometer (Beckman Coulter, Brea, CA) using control and labeled cell samples.

### In Vivo and Post‐mortem Murine MRI/MRS

#### Physiology

To ascertain the temporal effects of anesthesia, ISO was administered in three C57BL/6 mice (male [mean ± SD], 24.6 ± 3.6 g, weight range = 25.0–27.2 g). The mice were induced using 4% ISO, and were maintained for up to 95 min with 1.5–2.0% ISO, mixed in 100% oxygen.

All animals were placed in a specially constructed cradle (www.civm.duhs.duke.edu), and were allowed to breathe freely throughout the study. A homeostatically controlled hot‐air system was used to maintain mouse body temperature at approximately 37 °C. ECG and breathing rates were monitored using a gating system.^26^ Heart rates were maintained at 382–565 beats/min.

One additional C57BL/6 mouse (male, weight = 32.4 g) was euthanized with an overdose of ISO (5%) and was subsequently injected in the femoral area of the right hindlimb with labeled CDC cells, transferred to a butterfly coil, and then re‐imaged post‐mortem.

#### ^1^H Imaging

Axial cardiac ^1^H images (TR/TE = 2.98/1.49 ms; echo = 50%; flip angle = 15 °; NEX = 8; field of view [FOV] = 40 × 40 mm^2^; 10 slices; slice thickness (ST) = 1 mm; matrix = 128 × 128; acquisition time = 3.25 min; eight phase encoding steps per segment) were acquired with a 2D segmented *k*‐space pulse sequence. Correspondingly, post‐mortem skeletal muscle images (TR/TE = 2.33/1.18 ms; flip angle = 20 °; NEX = 1; 40 × 40 × 40 mm^3^; ST = 40 mm; and matrix = 128 × 128 × 128) were acquired with a 3D SPGR sequence.

#### ^19^F MRI/MRS

Double‐gated axial ^19^F images of the mouse thorax were acquired (TR/TE = 9‐17/4.6–8.4 ms; echo = 50%; flip angle = 50 °; NEX = 128–512; FOV = 40 × 40 mm^2^; 1 slice; ST = 10 mm; matrix = 32 × 32; BW = 2–4 kHz; acquisition time = 2.42 min; and 8 phase encoding steps per segment). Spectral selection of the ISO and NP label resonant peaks was achieved using broadband excitations and narrowband acquisitions centered on the resonant frequency of interest. For skeletal muscle imaging, the acquisition parameters were: TR/TE = 17/8.4 ms; flip angle = 50 °; NEX = 512; FOV = 40 × 40 mm^2^; 1 slice (coronal and axial views); ST = 40 mm; matrix = 32 × 32; BW = 2 kHz; and acquisition time = 4.5 min.

NP‐labeled cells (skeletal muscle areas) and the temporal evolution of ISO accumulation (30 successive spectra acquired and averaged over 5‐min intervals in thoracic areas) were monitored using nonlocalized MR spectra (TR = 20 s; flip angle = 90 °; NEX = 2–8; 512 points; BW = 10 kHz) within a temporal window of 35–90 min postinduction.

### Image Processing

#### Image and Spectral Analyses

^19^F images were imported and interpolated in ImageJ (NIH, Bethesda, MD) using bicubic splines to match the ^1^H matrix size. Thoracic ^1^H and ^19^F MRI were overlaid in ImageJ (opacity = 70%). For skeletal MRI, the outer hind limb boundaries were segmented in ImageJ by an expert user, and the segmented outline was overlaid on the ^19^F images. Spectra were processed in CSX (P. Barker, Johns Hopkins, USA) or using custom‐written IDL tools (Harris Geospatial, USA). For temporal ISO comparisons, peak integrals were estimated from gated, magnitude spectra.

#### Statistical Analyses

All results are reported as mean ± SD. The temporal constancy of the spectral areas for both ISO resonances is quantified using the coefficient of variability (CV = 100 × SD/Mean). Repeated‐measures, nonparametric, two‐tailed Student t‐tests, were also used (XLSTAT, Addinsoft, New York) to determine whether the ISO dose reduction (2.0% to 1.7%) caused significantly different changes in the areas of the spectral peaks (α = 5%).

## Results

**Figure 1:**
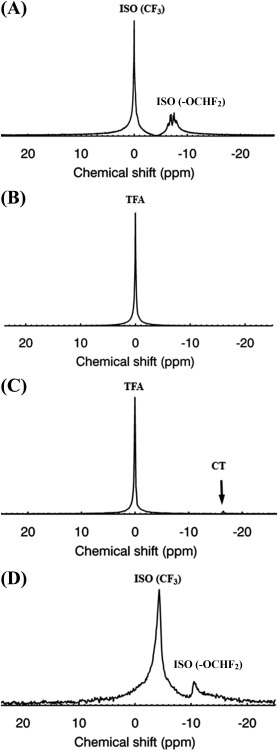
**A**: Spectrum of a 2 mL ISO phantom. **B**: Corresponding spectrum of a 75 mM trifluoroacetic acid (TFA) phantom (50 mL). **C**: Spectrum of a 25 mM TFA phantom in the presence of collagenase‐trypsin (CT) progenitor cells labeled using PFCE‐containing NPs (arrow). The NP resonance (‐16.25 ppm) is distinct from the TFA resonance (0 ppm). **D**: Typical spectrum of ISO signals (corresponding to the ‐CF_3_ and ‐OCHF_2_ moieties) in a mouse in vivo. The spectrum was collected and averaged over a period of 5 min. All spectra were acquired using a birdcage coil.

Typical nonlocalized pulse‐acquire ^19^F spectra of: (a) an ISO phantom, (b) a 75 mM TFA reference standard, (c) a 25 mM TFA phantom in the presence of an NP‐labeled cell phantom, and (d) ISO accumulation in thoracic areas in the in vivo mouse, are, respectively, shown in Figure 1A–D. For graphical presentations, all spectra are referenced to TFA at 0 ppm. Estimated in vitro linewidths are 92 Hz (ISO) and 210 Hz (75 mM TFA). More importantly, Figure 1D demonstrates that NP‐labeled CT cells exhibit a PFCE resonance at ‐16.00 ppm that does not overlap with the ISO resonances. Two distinct ISO resonances are observed in vivo (Fig. 1D) that, respectively, correspond to the ‐CF_3_, and ‐OCHF_2_ moieties.^14^, ^17^, ^19^ Noted also is a downfield spectral shift of the ISO resonances in vivo by approximately ‐4 ppm, and an increase in the spectral linewidth of the ‐CF_3_ peak to 500 Hz. The spectral separation of the two ISO resonances in vivo is ∼6.3 ppm (Table 1). Taken together, Figure 1C, D indicates that PFCE‐labeled CT cells exhibit spectral separations of ‐11.25 and ‐4.95 ppm from the ‐CF_3_ and ‐OCHF_2_ resonances of ISO, respectively.

**Table 1:** Extensively Used ^19^F Labels, Corresponding Cell Types, and Chemical Shifts with Respect to CFCl_3_ (and with Respect to TFA)^a^

^19^F Label	Cell Type	Chemical Shift with respect to CFCl_3_ [with respect to TFA] (ppm)
Poly(lactic‐co‐glycolic acid) perfluoro‐crown‐ether (PLGA‐PFCE)	Human dendritic cells (DC)/macrophages/monocytes/endothelial cells Cardiac progenitor cells (CDC, CT)	−91.8 [∼ −14.75–−16.00] (This study)
Perfluoropolyether (PFPE)	Primary T‐cells/neural stem cells/primary human DCs/	−58 [18.55] −90.7 [−14.55] −93.2 [−16.65]
Perfluorooctylbromide (PFOB)	Stem/progenitor cells/smooth muscle cells/macrophages	−63.7 [26.7] −81.82 [−5.27] −117.5 [−40.95] −122 [−45.45] −126.6 [−50.05]
Perfluorodecalin (PFD)	Monocytes‐macrophages	−27.4 [49.15] −32.0 [44.55] −38.7 [37.85] −49.6 [26.95] −98.2 [−21.65]
Trans‐bis‐perfluorobutyl ethylene (F‐44E)	Inflammatory cells – immune cells	9.1 [85.65] −23.7 [52.85] −33.4 [43.15] −35.0 [41.55]
ISO (ppm)		
‐CF_3_ moiety (in vivo)	‐	−80.55 [−4]
‐OCHF_2_ moiety (in vivo)	‐	−86.85 [−10.3]
TFA		
Aqueous solution	‐	−76.55 [0]
NaF		
Aqueous solution	‐	−121.5 [−44.95]
KF		
Aqueous solution	‐	−125.3 [−46.75]

a Also listed are the spectral shifts of the ISO moieties and of the aqueous reference standards TFA, NaF, and potassium fluoride (KF). Cardiac progenitor cells (CDC or CT) used in this study exhibit respective downfield shifts in the ranges of −10 to −11 and −4 to −4.95 ppm with respect to the two ISO moieties.

**Figure 2:**
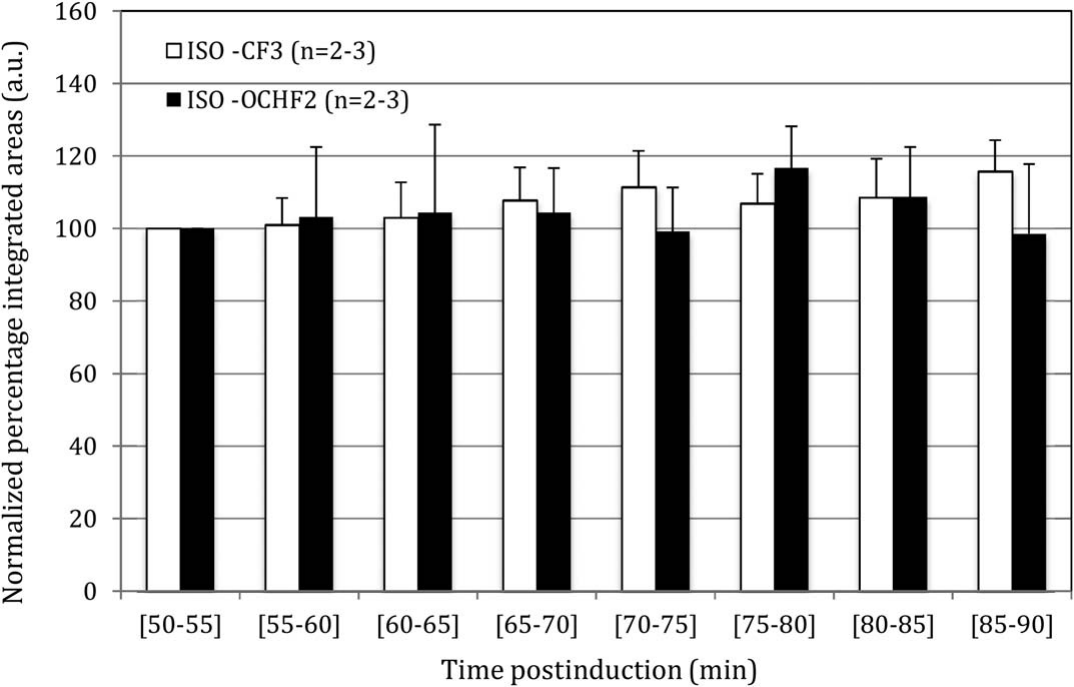
Quantification of normalized peak areas of the two ISO moieties at different time intervals postinduction in the three studied mice. The temporal constancy of the ISO signals was confirmed using coefficient of variability estimates. Statistically insignificant mean spectral area changes (*P* = 0.23; *P* = 0.71) were observed with respect to ISO dosage changes (α = 5%). Mean and error bars for the interval spanning t = 50–60 min reflect averages and standard deviations from two mice.

**Figure 3:**
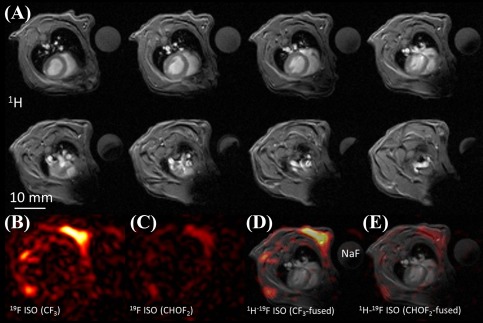
**A**: Multiple in vivo axial ^1^H MR images from one of the studied mice (eight out of ten acquired slices are shown, slice thickness = 1 mm). The ^1^H images correspond to the same volume as the ^19^F MRI. A cylindrical sodium fluoride (NaF) phantom is also visible within the field‐of‐view. **B,C**: Corresponding ^19^F images of the two ISO peaks (slice thickness = 10 mm. **D,E**: Merged ^1^H‐^19^F MRI of the reconstructed ^19^F images. For merging, a typical ^1^H scan was chosen within the acquired stack. ^19^F localizes in the skeletal muscle and fat areas of the thorax.

Figure 2 shows the in vivo temporal evolution of the nonlocalized, pulse‐acquire ISO signals (corresponding to the ‐CF_3_ and ‐OCHF_2_ moieties) at 5 min intervals from a typical mouse. To assess any changes in the in vivo ^19^F spectrum of ISO with time or dose, the areas of both ISO peaks were quantified using CSX, and the ISO dosage was decreased from 2.0% to 1.7% at t = 80 min postinduction (see Fig. 3).

The CV of the ISO peaks for each of the studied mice and for the entire cohort (during the time period of 50–90 min postinduction) were: (a) 6.4, 3.9, 3.2, and 3.9% (‐CF_3_), and (b) 11.2, 6, 8.5, and 6.4% (‐OCHF_2_).

Furthermore, no statistically significant changes in mean spectral areas of the ISO peaks (*P* = 0.23, *P* = 0.71) were observed pre‐ and postreduction of the ISO dose (t = 60–80 min versus t = 80–90 min).

In vivo localization of ISO was confirmed using ^19^F MRI, as shown in Figure 3. Axial ^19^F MR images (ST = 10 mm) of the two ISO moieties acquired using narrowband acquisitions are shown along with corresponding ^1^H images (only 8 of the 10 acquired slices, ST = 1 mm). To better demonstrate the anatomical localization of the ISO signal, merged ^1^H/^19^F images are also shown (Fig. 3C). For merging, a ^1^H image spatially located within the excited ^19^F imaging slab was selected. As it can be seen from the images, the ^19^F signal from the ISO peaks localizes in the skeletal muscle and possibly subcutaneous fat of the thorax, whereby both ISO resonances exhibit similar localization. The lack of ^19^F signals from the ventricular blood or myocardium is also noted.

**Figure 4:**
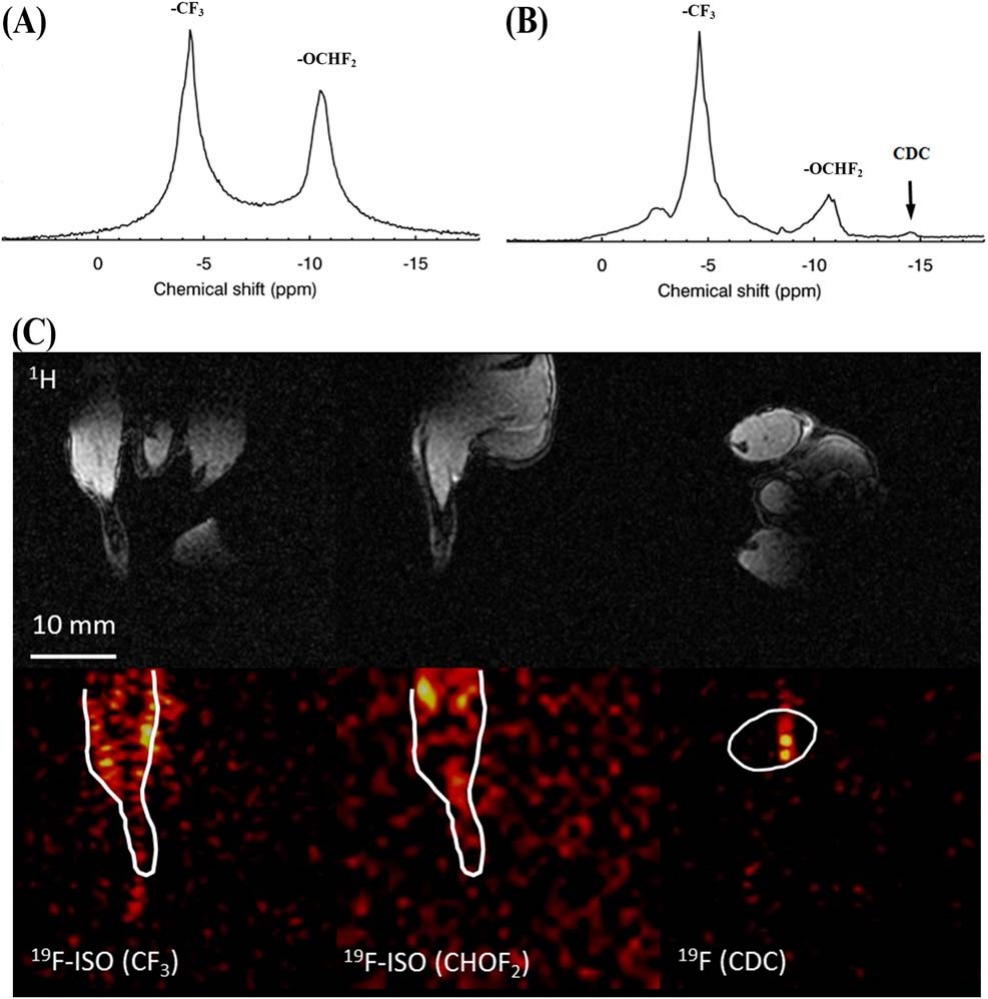
**A**: Nonlocalized, post‐mortem ^19^F spectrum from a male mouse. Experimental data collected in this study demonstrated ISO persistence over 7 h post‐euthanasia. **B**: Corresponding ^19^F spectrum of the femoral region of the right leg of the mouse following injection of ∼2.5 million cells cardiosphere‐derived cells (CDC) with NP labels. The arrow indicates the peak corresponding to the labeled progenitors, distinctly separated from the ISO peaks. ^1^H (**C**) and (**D**) ^19^F images of the ISO peaks were acquired using the butterfly coil. The spatial boundaries of the right hindlimb obtained from the ^1^H MRI are overlaid on the ^19^F MRI.

Figure 4 shows post‐mortem nonlocalized pulse‐acquire ^19^F spectra acquired immediately before and after injection of CDC cells labeled with PFC NPs. The spectra were acquired ∼7 h post‐mortem in a mouse that was injected in the femoral area of the right leg with ∼2.5 million CDC cells labeled with PFCE NPs. Indicated (arrow) is the resonance corresponding to the PFCE label, which does not overlap with the ISO resonances. Shown also are coronal ^19^F MR images corresponding to each of the two ISO resonances, and the corresponding ^1^H images. An axial ^19^F MR image corresponding to the ^19^F label is also shown where no observable contribution from ISO is observed.

## Discussion

This study demonstrates ISO accumulation in thoracic areas of the mouse. The results also demonstrate that the presence of ISO does not constitute a problem for the detection and quantification of PFCE in cardiac labeled cells.^20^ Herein, the two expected peaks of the NMR spectrum of ISO were observed both in vitro and in vivo.

ISO exhibited chemical shifts of ‐4.0 (CF_3_) and ‐10.3 ppm (‐OCHF_2_) in C57BL/6 mice in vivo with respect to the TFA resonance at 0 ppm, confirming previous reports in rabbits, and C57BL/6 and BALB/c mice.^14^, ^19^, ^27^ The downfield shift of ISO in vivo has been attributed to the chemical structure and the microenvironment in which ISO resides.^14^ It is also of interest to note that the chemical shift of the ‐CF_3_ resonance is either co‐resonant with (in vitro) or close to (in vivo) the TFA resonance. Consequently, TFA reference phantoms cannot be used in ISO/NP label studies because the signal overlap can lead to inaccuracies in calibration and quantification. Instead, NaF (‐44.95 ppm) or potassium fluoride (‐46.75 ppm) phantoms may be preferred because their chemical shifts do not overlap with those of the ISO moieties.

Substantial accumulation of ISO was observed in the live mouse as documented by ^19^F MRS. Prior work in vivo reports that the uptake and equilibration of ISO and sevoflurane in the brain is extremely fast (of the order of minutes),^15^, ^17^, ^28^ compared with the ISO release which appears to be extremely slow (of the order of 72–98 h).^14^, ^15^, ^28^

The results presented in this study show constancy and temporal stability of the evoked ISO spectra from anesthetized mice within time periods spanning 35–90 min postinduction. These findings are reinforced by the lack of statistically significant differences in the ISO signal amplitude following dose decreases. Additionally, the post‐mortem study confirmed persistence of ISO in thoracic and skeletal muscle areas for at least 7 h post‐euthanasia, consistent with prior literature.^14^, ^15^, ^16^ Thus, longitudinal studies with repeated imaging sessions may be affected by ISO accumulation. This issue has been overcome in prior work with the use of alternative anesthetics.^20^, ^29^

Noted also were significant increases in the linewidths of both ISO peaks attributed to either temporal, or physiologically dependent factors. From the physiological standpoint, the acquired spectra represent nonlocalized, spatiotemporal averaged signals that may be prone to: (a) beat‐to‐beat variations, (b) overall effects from ISO distribution (as a result of intraspecies variations in weight, body muscle/fat composition), and (c) heterogeneity of signal contribution arising from multiple organs and multiple T_2_ entities. From the technical standpoint, primary effects may be attributed to: (a) shimming, (b) B_0_/B_1_ effects (leading to flip‐angle dependencies and off‐resonance effects), and (c) the inability to resolve multiplet patterns from the respective spectral splitting of the two ISO moieties, ultimately leading to two distinct, broadened, peaks. Certainly, the in vivo ISO peak intensities are substantially larger than that of the PFCE spectral intensity arising from the labeled cells.

From the biophysical standpoint, it is expected that the observed linewidth changes are attributed primarily to shortened T_2_ or $T_{2}^{*}$ values, or to poor B_0_ homogeneity over the entire thorax or skeletal muscle areas. The adopted MRS approach and inferences drawn are certainly based on nonlocalized spectra, spanning the entire FOV of the RF coil. Correspondingly, T_2_ reductions are expected to be most likely an end‐result of ISO binding/complexation. The broad (nonlocalized) spectral peaks observed in vivo (compared with linewidths of 50–70 Hz reported by Wyrwicz et al.^14^ in rabbit brains) indicate the existence of either suboptimal shimming, or ultrashort T_2_ moieties and/or a heterogeneous, multifocal pattern of ISO accumulation in lipid, blood, and other tissue areas. Our results are in agreement with Xu et al.^15^ who have presented evidence for ultrashort T_2_ anesthetic moieties (<3 ms) in the brain in rats as a result of intracranial skeletal muscle/fat accumulations (14), despite the lack of significant lipid accumulations/contributions as reported in a recent study by van Heeswijk et al.^19^

Given the chemical shift difference between the ISO resonance of the ‐OCHF_2_ moiety with respect to the PFCE resonance of cardiac progenitor‐labeled cells, and in conjunction with the selective acquisition bandwidths used in ^19^F MRI, we have shown negligible ISO effects on the imaging of ^19^F signals arising from PFCE labels in vitro, and in post‐mortem skeletal muscle imaging in the mouse. Combined selective excitation/suppression schemes can be used to excite the PFC label and minimize baseline drifts due to possible contaminating effects from ‐OCHF_2_. This is particularly important given the multifold ^19^F signal difference of labels in cells compared with the ISO moieties, particularly considering the expected submillimolar label concentrations in cells.^29^, ^30^, ^31^ The downside of selective excitation and prepulse suppression is the prolongation of TE. Nevertheless, in the case of the PFCE NPs, such effects are anticipated to be minimal, given their long T_2_ responses in the range of 50–600 ms.^7^

However, with the use of other commonly used ^19^F labels, such as perfluoropolyether (PFPE), perfluorodecalin (PFD), trans‐bis‐perfluorobutyl ethylene (F‐44E), or perfluorooctyl bromide (PFOB),^23^, ^32^ there is a significant potential for overlap of these labels and the ISO peaks, thereby confounding imaging and quantification. Despite the availability of other anesthetics and the possibility to ameliorate potential interference issues with alternative injectable anesthetic regimens,^8^ achievement of a stable dose level over the prolonged periods of MRI/MRS (injections and top‐ups) is challenging.

Despite the technical complexity of this work, there are several limitations in the study design. For example, despite the consistency of the effects of ISO observed in the male mice of this study, and in preliminary studies in female mice (results not shown), the sample sizes were small. Therefore, possible sex differences in the ISO effects need to be accounted for in large‐sample studies. Furthermore, the reported effects of temporal accumulation of ISO and its biodistribution, as well as the spectral response of the PFCE NPs must be assessed in models of pathology, including myocardial infarction and skeletal myopathies. Of more importance may be also the generalizability of the study and its extension to larger animal models (e.g., rats or pigs) in larger bore, lower‐field magnets. However, migration of the work may impose further challenges in terms of the separability of the ISO and PFCE NP spectra. Thus, a more appropriate study design may also include the characterization of the T_1_ relaxation values for both anesthetic moieties, and the selective inversion of the undesired spectral peaks in conjunction with the selective excitation of the peaks corresponding to the labels.

In conclusion, accumulation of ISO was observed in the mouse with signals that are temporally stable over 90 min post‐induction, as documented by ^19^F MRS. The temporal persistence of ISO spans multiple hours. Given the spectral separation of the ISO peaks from the PFCE resonance, and the selective acquisition bandwidths often used in ^19^F MRI, the anticipated signal overlap/contamination effects of ISO on PFCE labels are minimal, but may be more prominent for PFPE or PFOB labels. Correspondingly, ISO still remains the primary choice for ^1^H and multinuclear imaging studies.
